# Data condensed synthesis regarding kinesiotherapeutic procedures used in spasticity therapy

**Published:** 2014-09-25

**Authors:** E Moraru, G Onose

**Affiliations:** * Neurological Rehabilitation Clinic HWK I, Bad Zwesten, WWK – Hessen, Germany; ** "Carol Davila" University of Medicine and Pharmacy, Bucharest; “Bagdasar-Arseni" Clinical Emergency Hospital; Neuromuscular Recovery Clinic, Bucharest, Romania

**Keywords:** upper motor neuron syndrome (UMNS), muscle paresis, spasticity, kinesiotherapeutic procedures, kinesiotherapy

## Abstract

Abstract

Spasticity represents an important feature of the upper motoneuron syndrome (UMNS). The clinical signs, such as the abnormal movement models, the unwanted muscular co-contractions, the muscular and joint rigidity with a consecutive deformity can be signs of spasticity and, also of upper motoneuron lesion. The different therapeutic options applied in the management of spasticity are a basic component of UMNS treatment scheme. This article presents the main kinesiotherapeutic procedures used in spasticity therapy.

## Introduction

The Upper Motor Neuron Syndrome (UMNS) can appear as a result of the lesion of the central nervous system (CNS). According to the type of the lesion, the area and the evolution in time, this has different symptoms. An important feature is spasticity and “according to the definition given by Lance in 1980, it represents a motor disorder characterized by a velocity-dependent increase of the muscle tone (tonic stretch reflexes) resulting from hyperexcitability of the stretch reflex" [**1**]. The researches undergone have developed different methods of intervention in spasticity management, the therapeutic options being a basic component of UMNS treatment scheme [**3**]. Recent studies have proved that the nervous system can be remodeled through neuroplasticity during life, from a physiological point of view and after having suffered from lesions, through processes of re-learning, including through experience, regained as an answer to the activity which is daily repeated, training or/and frequent iterative practicing [**2,3,6**]. Moreover, neuroplasticity refers to changes in the neurotransmission activity at the cellular level, having as a result “the modeling of reflex activity" [**2**]. 

 Some of the kinesiology treatment options are the following: stretching exercises, associated or not with orthotic equipment (by specific bandages or splints, possibly dynamic, or even rarely, by immobilization in serial casting), connected therapeutic exercises such as occupational therapy (including neurodevelopment training). Other procedures that can be applied are the physical-nonkinetic ones such as electrical stimulation (Transcutaneous Electrical Nerve Stimulation – TENS – or functional – FES), EMG biofeedback, galvanotherapy, magnetotherapy, phototherapy, hydro-, thermo-/ cryotherapy, ultrasound therapy [**4,5**].

 Taking into account the limitations of the editorial space, this article contains condensed information regarding the kinesiotherapy demarches. 

Kinesiotherapy procedures based on passive mobilization 

Stretching exercises 

 The exercises included in the stretching therapy are methods of treatment to which both the kinesiotherapists and the occupational therapists turn to. Preserving functional muscle length involves both passive and active stretching. Preservation of the extensibility of soft tissue, in particular calf muscles and rectus femoris, is critical to the patient's ability to stand up, walk and stair walk [**3**]. Stretching is defined as a process of elongation (of a structure – a tissue – which is potentially extensible) [**6,7**]. The intimate action mechanism is not fully clarified, neither are the theories which are most frequently used, unanimously accepted. In addition, the basis of this “influencing-training" process is represented by the mechanical modifications or/ and the thixotropic modifications connected to the lysis of some functional, residual connections, among the actin molecules and the myosin molecules at the musculotendinous level. On the other hand, “the repetitive activation" would lead to an inactivation of the calcium channels at the presynaptic level and to modifications in the “synaptic transmission efficacy, associated with changes in the neurotransmission activity at the cellular level", having as a result the “adaptation" (disfacilitation) of the reflex activity" [**9**]. 

 For example, a significant effect would appear in the spastic hemiparesis in over 30 repetitive movements of the elbow. It is considered that the minimum duration of an efficient stretching is of thirty seconds, but there is also the idea that as long as a stretching is greater the more efficient it is. The principle of “repeated adjustment" and stretching can be found in Bobath’s “Neurodevelopment Technique" as well as in the proprioceptive neuromuscular facilitation techniques [**9**]. It is considered that the hypertonic reduction effect would last for a few hours (enough, at least to doing the exercises for the improvement of the voluntary motor control, coordination, balance, walking, ability, force and contraction tension). 

 There are many methods of application for the stretching therapy, but most often, the therapist does the movements manually. The use of a mechanic device, such as a dynamometer (Cybex) for stretching is an alternative to the manual maneuvers. The use of some mechanic devices allows a better standardization for treatment and research protocols. Dynamometers such as Cybex, Kin-Com and Biodex, control devices based on feedback technique are used more and more in order to make well-controlled and standardized stretching maneuvers. It was noticed that the force of the paretic muscle has increased during the treatment [**5**]. 

 The tilt table has been widely used to fight the effects of spasticity at the level of the legs joints, in the management of stiffness/ joint deposturising. When they are correctly used, numerous joints, the one of the knee, hip, etc., even the joints of the arms, can be simultaneously treated in specific conditions. The stretching intensity can be controlled by the modification of the angle to which the table is tilted. By increasing the tilt angle and bringing the patient to an almost vertical position, the weight/gravitational force acts on carrying myo-tendinous structures. The benefic actions of this method mainly include the following: posture ameliorations, a better bronchial cleansing, pro-gravitational amelioration of the urinary drainage and the intestinal transit [**3,4**].

 Another method, which allows a functional position is the standing frame [**3,4**]. The standing frame permits a more active participation of the patient, being also used as a physical support during some subsequent stages of the treatment. For example, it is used at the end of a recovery kinesiotherapy session, to put the patient in a certain position so as to be able to perform occupational and recreational activities. Paradoxically/ dialectically, it is advisable on one side for the patients who are biologically stable, have an adaptive tolerance to posture and related hemodynamic modifications, as well as on the other side, in paraplegic patients without any motor control recovery perspectives of the affected area. Regarding these aspects, we further quote an important conclusive synthesis: "Basically, the progressive tilt table is used in severe cases (from the point of view of the general neurologic status or/ and the general biological state, endurance included) but also in the precocious training for the realization of the progressive gravitational loading and the practicing of the walking alternative sequences". The vertical position, walking included, assisted by these devices, mainly target the spasticity elimination, having the following beneficial results: 

 - the stimulation of the central movement generator pattern (CMGP) at the medullary level, together with the proprioception and the sensitive/ sensorimotor feedback 

 - the axial balance and coordination improvement 

 - the improvement of the bone trophic 

 - the facilitation of the urine gravitational drainage and the bowel transit 

 - the improvement of the image/ self-esteem and the quality of life (QOL), offered by the orthostatic position, which is specific for human beings" [**4**]. 

**Fig. 1:**
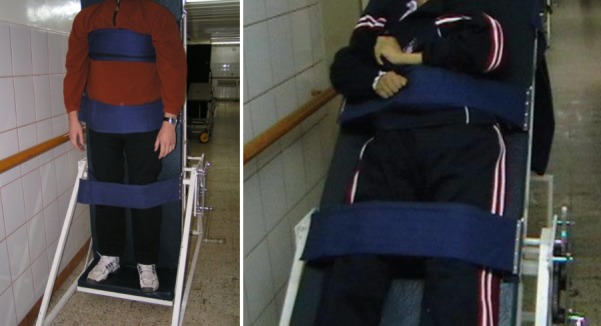
Tilt table with progressive verticalization

**Fig. 2:**
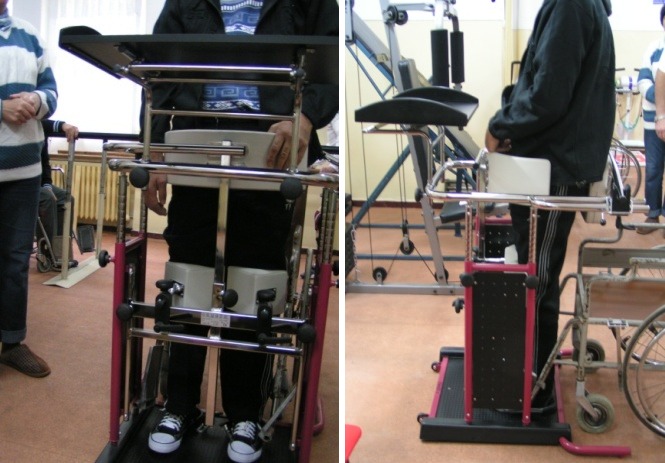
Standing frame

Orthotics

 An important device applied in the rehabilitation therapy is the orthosis. It is an externally applied device used to modify functional and structural characteristics of the neuromuscular system. In order to prescribe an orthosis the following elements should be taken into consideration: 

 - the identification of biomechanical mechanisms which subscribe to the walking dysfunction, 

 - the relative contribution of the active and passive components of the tissues,

- the orthoses should not allow an unwanted movement and should not restrict a wanted movement either. [**11,12**]. When the plantar flexor muscles are spastic or/ and there is a contracture, little doses of botulinum toxin or repeated “casting" combined with the training of the walking, can be necessary [**5,8,10**]. 

 The dynamic splints are devices that contain two components: active and passive [**3**]. These devices allow the functional movements outside the spastic spectrum. The dynamic splints, which are usually used, include Dynasplint, SaeboFlex and other devices made by using a combination of springs and pulleys, which assure a dynamic component. This type of splints can also be projected and manufactured in order to allow movement in case of certain joints without external assistance. 

 Lycra buttocks are projected to produce a continuous stretching of the muscle/ spastic muscular group, when they are worn for a couple of hours per day. Moreover, lycra buttocks had quick effects at the level of the fist (basically the RCC joint) and fingers, in the case of hemiplegic patients. These buttocks are made up of segments, which are tensioned in the wanted direction, so that the elastic material exerts a continuous stretching in a certain direction. Although they are not yet used on a large scale, the buttocks have gained more ground in the recovery units [**3**]. 

 The serial casting (SC), which is also called inhibitory casting, has been used for a couple of decades in spasticity management. It has been described for the first time in 1960, in patients with cerebral palsy. After a long period of immobilization, the risk of deep venous thrombosis and osteoporosis can appear. 

 The elbow joint, the joints of the toes and ankle are the ones which benefit most often from this treatment [**5,11**]. SC was used especially after the appearance of muscular and joint rigidity. Injections of botulinum toxin may also be effective in decreasing stiffness where it is extreme and accompanied by reflex hyperactivity. Today, SC is rarely used, due to other therapeutic procedures which can prevent the muscular rigidity. It is ideal that spasticity is treated on time, before the appearance of fixed contracture and consecutive deformity. The repeated application of the casting, while the joint is as stretched as possible, helps in the improvement of the motion range, the functionality and the reduction of pain. The final casting can be kept in order to be used as a position maintenance orthosis. Local therapies with botulinum toxin and serial casting have been successfully used in children suffering from cerebral palsy [**8,10**]. 

Kinesiotherapy procedures based on active mobilization

Exercises are an essential part of the rehabilitation process of UMNS patients. Studies favor the inclusion of therapeutic exercises, such as the non-resistant pedaling and the strength exercises in the recovery program of the spasticity patients. 

Non-resistant pedaling 

 For example, the effects of pedaling on spasticity by measuring the H reflex (reflex reaction of muscle that appears after electrical stimulation of sensory fibers in their innervating nerves) and the MAS (Modified Ashworth Scale) have been evaluated in twenty-seven patients with multiple sclerosis [**13**]. The patients have done a pedaling exercise for 20 minutes, on a cycle ergometer, without any resistance. The measurements have been made before the starting of the exercise and at ten, thirty and sixty minutes after it. The results demonstrated reductions both in MAS and in the Hmax/ Mmax rate at each experimental interval of time. The authors have concluded that pedaling on a non-resistant bicycle has been beneficial for the reduction of spasticity of the treated patients. 

 A small study with nine post-stroke patients analyzed the impact of cycle ergometry (repetitive arm cycling) on spasticity and performance at the level of the arm [**14**]. A decrease of spasticity measured by AS at the level of the flexors and extensors of the elbow and an increase of the maxim active extension at the level of the biceps has been registered after the patients have pedaled for five days per week for 3 weeks. In addition, an increase in the active movement area of the elbow and of the muscular force has been registered. 

Force exercises 

In a synthesis of seven random tests that examine the effects of the force exercises in post-stroke hemiplegic patients, it was noticed that intense force training does not have negative effects on spasticity and that the progressive resistance training has a positive impact on all the vital functions. In addition, the increase in the muscular force at the level of the feet (flexor/ extensor muscles of the knee, plantar/ dorsal flexor muscles of the foot) improves the walking ability and the balance [**15**]. The studies which have evaluated the effects of the force exercises on post-stroke patients have concluded that they help in increasing the muscular force, the functional capacity and do not emphasize spasticity [**3,15**]. 

Occupational therapy

 The following can be mentioned as areas of interest for occupational therapy (OT): skills (walking, communication, interaction), the use of some objects and the understanding of their characteristics, the therapy of the deficits of perception and cognition, DLA (daily life activities)/ DLIA (daily life “instrumental" activities), education, working, playing, social participation connected to the cultural, spiritual, personal context. The performances which are necessary to some normal activities are the ones in the walking field (coordination, balance, objects manipulation), sensitive-sensorial and perception field (tactile, visual, auditory), cognitive field (thinking, selection, organization, creation), emotional regulation field (anger and frustration management, coping strategies), communication and social life field (gestures) [**16**]. 

 The term “normal movement" is used to identify a specific coordination behavior used by people to perform a certain activity. Normal movements use models of the biomechanical and kinesiology activity adapted to the human body. This way, the normal movement is possible due to the interaction between the musculoskeletal and nervous system. The ergotherapist’s role is to help in the process of learning new ways of performing an activity, for example by splitting the movement in smaller component sequences, which will be used until the movement as a whole is learnt. OT can also address the prevention of the mechanic movements blocks, as a result of the tissue rigidity with a possibility of a fixed contraction of the limbs [**3,16**]. When spasticity and abnormal movements are present, therapy also has a role in restoring the normal muscular tension and in reeducating the function of the movement. 

## Conclusions

Spasticity represents an important issue in neurorehabilitation. Both the multidisciplinary therapy and the continuous evaluation of the spasticity are essential in defining and acquiring the improvement of patient function. Some patients can be effectively treated with a combination of kinesiology measurements as well as with local pharmacological agents and oral anti-spastic drugs. Moreover, an adequate hygiene can reduce the external stimuli that exacerbate the muscle tone. The adequate management of the complex problem of spasticity needs a large expertise from the team and includes physicians in the field of rehabilitation, neurologists, orthopedic surgeons, neurosurgeons, kinesiotherapists, occupational therapists, specialists in orthotics, nurses in the field of physiotherapy and healthcare.
